# 3D Segmentation of Multi-contrast Cardiac Magnetic Resonances With Topological Correction and Synthetic Data Augmentation

**DOI:** 10.1007/s10439-026-04115-8

**Published:** 2026-04-18

**Authors:** Ricardo M. Rosales, Manuel Doblaré, Esther Pueyo

**Affiliations:** 1 Aragón Institute of Engineering Research (I3A), Zaragoza, Aragón Spain; 2 Aragón Institute for Health Research (IISA), Zaragoza, Aragón Spain; 3 University of Zaragoza, Zaragoza, Aragón Spain; 4 CIBER-BBN, Zaragoza, Aragón Spain

**Keywords:** Cardiac magnetic resonance, Convolutional neural networks, Generative adversarial networks, Persistent homology, Synthetic data augmentation, Topological correction

## Abstract

**Purpose:**

Automatic segmentation of cardiac magnetic resonance (CMR) images improves the evaluation of heart structure and function, helping clinical diagnosis and the generation of in silico models. Recent advances have introduced synthetic augmentation (SA) using generative adversarial networks (GANs) and topological correction (TC) via persistent homology to enhance segmentation with convolutional neural networks (CNNs). However, their combined effectiveness remains unexplored. Here, we extend and systematically evaluate these techniques, individually and in combination, for the first time in the context of three-dimensional (3D) CMR segmentation across challenging multi-vendor, multi-center, multi-class, and multi-contrast data sets.

**Methods:**

Data involved anisotropic, topologically inconsistent cine and late gadolinium-enhanced (LGE) CMRs, and isotropic, topologically consistent ex vivo CMRs. Topological priors were defined in each data set from ground truth label (GTL) assessments, and TC was applied by retraining the baseline 3D CNN with a loss function accounting for topological discrepancies. For SA, deformed GTLs were used to generate synthetic images using trained 3D GANs.

**Results:**

Consistent segmentation improvements were observed for the ex vivo data in both overlap with GTLs and topological precision when applying TC and SA individually and in combination. Notably, an enhanced identification of the infarction was obtained when SA and TC were used in the LGE data. Overall, SA increased the predictions overlap with GTLs, while TC reduced the topological discrepancies across all data sets.

**Conclusion:**

TC and SA demonstrate strong potential for improving 3D CMR segmentation on complex, real-world data sets, especially when topologically consistent data are available for training.

## Introduction

Cardiac magnetic resonance (CMR) is one of the most widely used imaging techniques to non-invasively assess the structure and function of the heart. There is a wide range of CMR modalities that allow the evaluation of different cardiac characteristics. The CMR modalities include cine and late gadolinium-enhanced (LGE) sequences, which are commonly used in clinical settings, as well as diffusion-weighted (DW) imaging, which is frequently employed in research for ad hoc modeling and simulation pipelines. Cine CMR consists of in vivo imaging of the cardiac cycle with configurations showing long-axis (LAx) planes (2-, 3-, and 4-chamber views) and a stack of short-axis (SAx) planes of the heart[1]. LGE-CMR is considered the standard method for the individualized characterization of acute and chronic myocardial infarction (MI), by in vivo intravenous administration of gadolinium contrast agents that travel from the injection site to ventricular blood pools [2]. DW-CMR is primarily conducted ex vivo to measure three-dimensional (3D) water diffusion, enabling the microstructural characterization of the heart and the estimation of cardiac fiber orientation [3].

Segmentation of CMRs is central to many clinical studies and modeling pipelines for identifying patient-specific features across cardiac pathologies. Traditionally performed manually by expert clinicians, this process is highly time- and resource-intensive. Deep learning (DL) has demonstrated the ability to achieve accurate and efficient automated segmentation, enhancing patient screening, anatomical delineation, and prognosis. Notably, convolutional neural networks (CNNs) have achieved clinician-level performance in numerous biomedical segmentation tasks [4].

For CNN to provide good performance, a large amount of research has been conducted to overcome cumbersome aspects specific to medical segmentation, such as data scarcity and high data variability, the latter caused by the multi-center, multi-vendor, and multi-type nature of images [1, 5, 6]. Cardiac segmentation is especially challenging, as contraction-relaxation cycles and respiration can lead to substantial motion artifacts in the in vivo CMRs, such as LAx inter-slice misalignment [7]. To improve cardiac image segmentation, many CNNs using per-slice (2D), multi-slice (2.5D), or volumetric (3D) images with different pre- and postprocessing techniques have been evaluated. Preprocessing usually involves image intensity normalization and spatial cropping, padding, and resampling. Postprocessing can include multi-segmentation averaging, smoothing, all-but-largest connected component suppression, and geometrical deformation guided by high-resolution atlases, among others. Moreover, model overfitting can be prevented by pre-training the CNN with similar data or employing standard data augmentation (DA) techniques, such as variations in brightness, random contrast, and rigid and non-rigid geometrical transformations [8].

### Generative Adversarial Networks

Generative adversarial networks (GANs) allow data generation by learning the real data distribution in a two-player minimax game [9]. Simply, a generator (G) learns a generative model of real data by being trained to fool the discriminator (D), which is simultaneously trained to accurately distinguish between real and G-created synthetic data.

Recently, GANs have shown promising results in biomedical DA by increasing the amount of training data with new synthetic samples [10]. Using GAN-based DA, Frid-Adar et al. [11] improved lesion classification on liver computed tomography, and Sandfort et al. [12] achieved a marked decrease in multi-organ volume prediction errors for non-contrast computed tomography images. In CMR, Al Khalil et al. [13] developed a synthesis module combining variational autoencoders and a GAN to generate synthetic SAx and LAx images. Similarly, Lustermans et al. [14] demonstrated that incorporating synthetic GAN-generated LGE SAx images enhanced MI segmentation by 3% and 6% when a single CNN and a cascade pipeline were used, respectively.

In all these works, conditional GANs (cGANs) were used, such as pix-2-pix [15] and cycleGAN [16], for paired and unpaired image-to-image translation, in addition to the spatially adaptive (de)normalization (SPADE) GAN [17] for semantic to real image translation that avoids semantic information vanishing. In cGANs, synthetic data generation and discrimination are conditioned by an input image, and the resulting G is capable of mapping data from this input image domain to another.

### Anatomic Consistency and Persistent Homology

Different segmentation methods have been proposed to achieve consistent anatomical outcomes. CNNs generally involve the minimization of pixel-wise loss functions, which hinders the learning of anatomically coherent structures [8]. In [18], convolutional autoencoders were used to generate low-dimensional representations (shape codes) of predictions and ground truth labels (GTLs) of cardiac computed tomography images, which allowed the addition of a shape-based regularization term in the objective function. Moreover, the segmentation of a cine-CMR slice can be anatomically constrained by previously segmented slices, namely, by combining the contextual and current latent spaces of 2D inputs from the same 3D image, as proposed in [19]. These methods were based on the strong assumption that anatomical definitions can be implicitly encoded in latent representations whose blurry nature leads to difficulties in the encoding interpretation and segmentation supervision [20]. In addition, these strategies rarely take advantage of the precisely defined 3D cardiac topology, which remains approximately steady even in a broad spectrum of pathologies.

Automatic topological simplification can help to explicitly clean topological noise from the data under study [21]. Initially, a 2D or 3D image can be represented as a cubical complex, which is a set of points, segments, squares, cubes, and/or their hyper-dimensional counterparts. The topology of the cubical complex is determined by the calculation of its Betti numbers B$_\text {0}$, B$_\text {1}$ , and B$_\text {2}$ whose values indicate the presence of connected components, tunnels, or voids in the topological space, respectively. In the context of persistent homology (PH), the Betti numbers are calculated for different cubical complexes obtained by the modification of a single variable, a procedure known as filtration. As an example, consider $\hat{{\textbf {Y}}}$ being a 3D probabilistic output of a CNN performing binary segmentation. $\hat{{\textbf {Y}}}$ has shape $i\times j\times k$ and per-voxel probability $\hat{{\textbf {Y}}}_{ijk} \in [0,1]$ of belonging to the foreground class. In this case, the filtration consists in the variation of a threshold p∈[0,1] to obtain level sets ${\textbf {S}}(p)$ that contain all voxels with $\hat{{\textbf {Y}}}_{ijk} \ge p$. During this filtration, the creation and destruction of topological entities occur and can be encoded in a birth–death diagram, as can be seen in the upper panel of Fig. 4c. The topological entities with longer lifetimes, that is, located far from the diagonal in Fig. 4c, correspond to persistent meaningful topological characteristics of $\hat{Y}$ [21, 22].

The differentiable properties of PH can be used, in combination with some prior topological knowledge of a cubical complex, to drive DL algorithms to achieve topologically consistent results [23]. Clough et al. [24] showed that the addition of a topological loss improved the classification of noisy handwritten digits and the 2D CMR segmentation of the left ventricle (LV) myocardium. Furthermore, Byrne et al. [20] reported a slight enhancement of multi-class segmentation in the context of 2D medial SAx and 3D whole heart images.

### Study Contributions

This work advances the state of the art by providing new insights into the performance of cutting-edge GAN-based synthetic augmentation (SA) and topological correction (TC) for 3D segmentation of complex, real-world CMR data sets. Modern multi-vendor, multi-center, and multi-class CMRs are considered for segmentation of cine and LGE images, while a manually segmented ex vivo data set is used for evaluation in a more topology-controlled set of samples. CNNs and GANs are trained on 3D data for segmentation and SA, respectively, and a topological loss is selected to achieve more anatomically coherent segmentation [20]. Importantly, we propose a selective application of TC guided by a prior topological assessment of ground truth labels (GTLs) to prevent prediction degradation due to data set-specific topological inconsistencies, as previously reported in 3D CMR segmentation [20].

## Materials and Methods

**Fig. 1:**
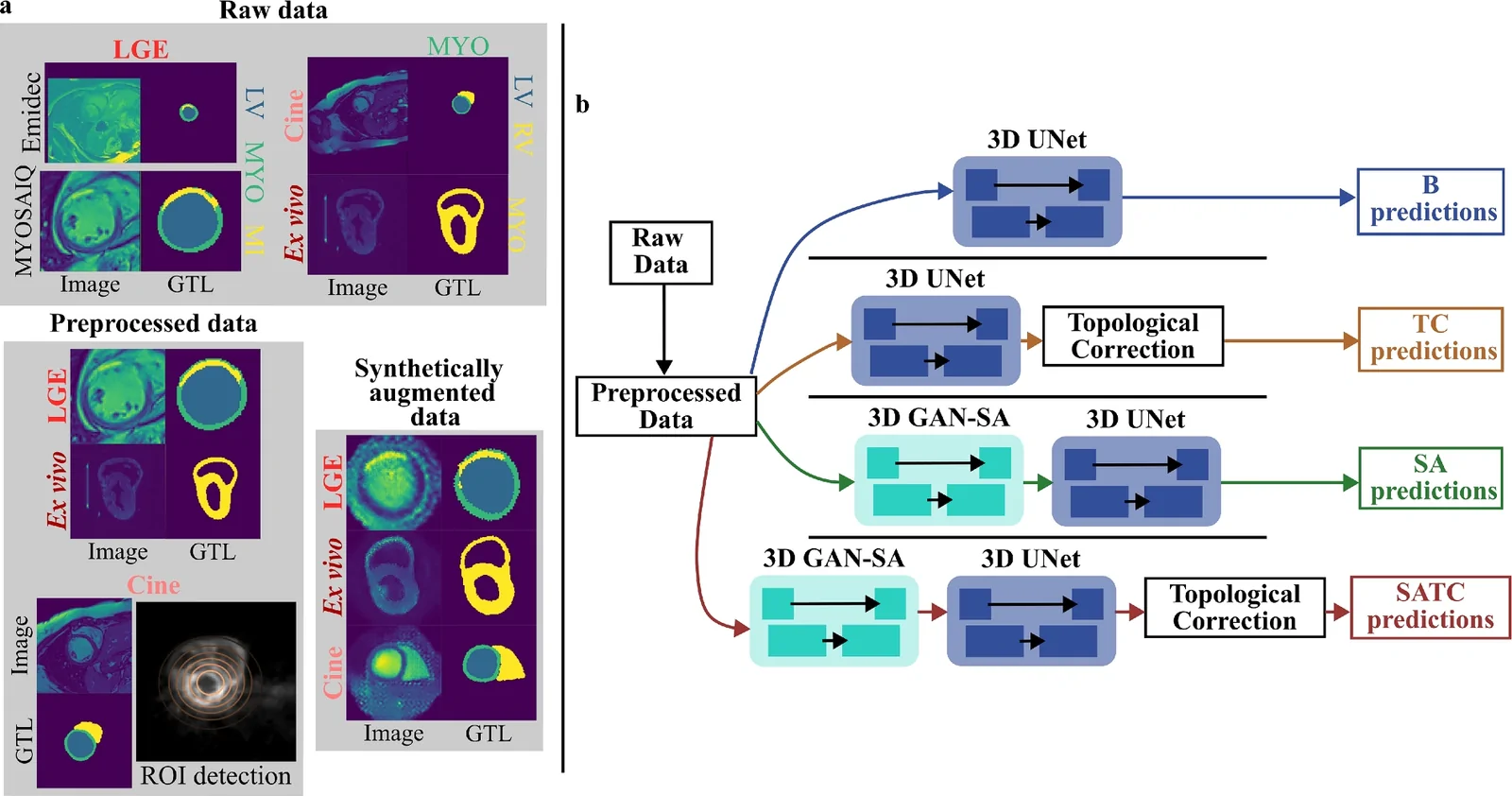
**a** Examples of raw, preprocessed, and GAN-augmented CMR data. **b** Simplified, schematic overview of the segmentation pipeline, illustrating the steps applied independently to each of the three CMR data sets to obtain the final predictions. *LGE* late gadolinium-enhanced, *LV* left ventricle, *MYO* myocardium, *RV* right ventricle, *MI* myocardial infarction, *GTL* ground truth label, *ROI* region of interest, *3D* three dimensional, *GAN* generative adversarial network, *B* baseline, *TC* topological correction, *SA* synthetic augmentation, *SATC* synthetic augmentation and topological correction, *CMR* cardiac magnetic resonance

The segmentation pipeline (Fig. 1b) consisted of preprocessing, GAN-based SA, topological evaluation, training, inference, and postprocessing. Each component is described in detail below.

### Data Sets

Cardiac microstructure, ventricular geometry at end-diastole (ED), and MI identification are key features for clinical diagnosis and computational modeling. Therefore, publicly available data sets representing these data types were selected.

#### Cine CMR

The open access M&Ms challenge data set contained 345 human cine CMRs acquired in three different Spanish and German healthcare centers with 4 different scanner manufacturers. Data comprised images of healthy volunteers and patients with hypertrophic and dilated cardiomyopathy [1]. The labeled data included contours for the LV myocardium and the LV and right ventricle (RV) cavities (see Fig. 1a). Unlabeled data were not included in the analysis.

#### LGE-CMR

The Emidec [2] and MYOSAIQ [6] data sets containing human LGE-CMRs were combined here. The Emidec data set originally consisted of 150 samples. However, the 50 test samples were not considered as GTLs were not available. For simplicity, only MI cases without microvascular obstructions from both data sets were used. In addition, a sample of the MYOSAIQ data with an extremely large LV was discarded. This selection resulted in a multi-vendor, multi-center, and multi-class LGE data set comprising 288 samples from MYOSAIQ and 27 from Emidec, with labels for the LV cavity, LV myocardium, and MI region (Fig. 1a).

#### Ex Vivo CMR

A publicly available data set from the Stanford Cardiac MRI Research Group containing 7 ex vivo porcine DW-CMRs was used [25]. The T1- and T2-weighted sequences and zero gradient (b0) images from the DW-CMR were included in the analyzed data set, which comprised 21 samples (no diffusion data were used). The manual segmentation for generating topologically consistent GTLs (Fig. 1a) was enabled by the high resolution and motion-free nature of the data. The GTLs included the biventricular myocardium from base to apex without the outflow tracts, which gives a clear topology of one connected component (B_0_ = 1) without tunnels (B_1_ = 0) and voids (B_2_ = 0).

### Data Preprocessing

Due to the differences among the three data sets, specific preprocessing steps were required.

#### Cine CMR

Initially, small spurious islands were removed from the GTLs in a label-wise manner. The overall physical extent and voxel-wise physical resolution varied across the data set. These differences arose from variations in the number of voxels per spatial direction, the voxel space, and the physical length represented by each voxel in each direction, i.e., the voxel-wise resolution (also referred to as voxel-wise spacing). Consequently, the physical space represented by each voxel was inconsistent between samples. This is problematic because standard CNNs operate on fixed grids and do not inherently account for physical spatial properties. Following state-of-the-art practices, we performed resampling to homogenize the physical space represented by each voxel [4, 13]. We adopted a plane-specific resampling strategy due to the anisotropic resolution of the data (high in the SAx plane and low out-of-plane). For the high-resolution SAx planes, volumes were resampled to the cohort-wide median voxel-wise spacing. Linear interpolation was applied to the images, and nearest-neighbor interpolation was used for the GTLs. For the low-resolution LAx planes, volumes were resampled to the $10^{th}$ percentile voxel-wise spacing. In this case, nearest-neighbor interpolation was used for both images and GTLs. Using nearest-neighbor interpolation for the GTLs prevented the creation of spurious voxels with non-existent intermediate class values. For the images, this out-of-plane strategy mitigated common interpolation artifacts. Specifically, it avoided generating artificial intermediate slices that could lead to abrupt and unrealistic contour variations between adjacent slices [4, 13].

As illustrated in the top Fig. 1a, the SAx cine-CMR images depict several structures in addition to the heart, making the detection of a region of interest (ROI) necessary. In cine CMR, the time evolution of the cardiac cycle and the fundamental spatial frequency generated by cardiac beating can be used in a Fourier-based technique to determine an ROI [26, 27]. First, a centered crop equivalent to 80% of each SAx plane dimension was applied. In other words, an image of 100×100×10 voxel space resulted in an image of 80×80×10 voxel space with equivalent center. The cropped 3D images were used to perform a per-slice 2D Fourier transform, and only the first harmonic was retained to compute the inverse transform. The resulting 3D array was subsequently processed by applying median filtering, normalization, and removal of all values below 5% of the maximum intensity threshold. In some samples, the SAx borders exhibited prominent first-harmonic components, which interfered with the Fourier-based ROI determination. To address this issue, a 2D image capturing the combined per-slice first-harmonic motion information was generated by summing the stack along the LAx direction, as illustrated in the ROI detection image in the bottom Fig. 1a. Iteratively, a Gaussian distribution (in orange in the bottom Fig. 1a.) was fitted to this motion image and the boundaries corresponding to less than 5% of its height were removed until the center of the Gaussian distribution changed position by less than one pixel.

Once homogeneous voxel-wise spacing was achieved and the ED samples were SAx-cropped to their corresponding ROIs, zero-padding was applied to ensure a common target voxel space across all samples. The definition of this target voxel space followed three criteria: (i) the lower bound of this target voxel space was determined by the minimum per-dimension voxel space identified in the preprocessed data set; (ii) high voxel spaces led to higher memory load with the addition of irrelevant background information; and (iii) selecting a target voxel space with dimensions divisible by 2 facilitates the design of convolutional layers within the network architecture. Consequently, the final preprocessed ED cine data set comprised 320 samples with a uniform voxel-wise spacing of 1.25×1.25×8.8 $\textrm{mm}^{3}$ and a voxel space of 160×160×20.

#### LGE-CMR

The MYOSAIQ samples were already cropped and LV-centered, as shown in Fig. 1. As the Emidec SAx plane was centered in the LV, we cropped the voxels equivalent to 60 mm in each of the 4-sided SAx images. Due to their anisotropic resolution (high in the SAx plane and low out-of-plane), the images and GTLs of both data sets were resampled as previously described. In addition, the target voxel space was selected with the same criteria presented for cine CMRs and achieved through zero-padding or cropping. The preprocessed data set comprised 315 samples with common 1.56×1.56×5 $\textrm{mm}^{3}$ voxel-wise spacing and 64×64×24 voxel space.

#### Ex Vivo CMR

This data set was already consistent in terms of voxel-wise spacing and voxel space. It was the one out of the three with the lowest variability in its properties because it was acquired from a single-vendor, single-center study. Here, we only checked that myocardial GTLs were composed of a single connected component (B_0_ = 1, B_1_ = 0, B_2_ = 0; see “Ex Vivo CMR” Sect.) without any resampling, cropping, or padding. As the data were acquired ex vivo, isotropic voxel space and high resolution were possible on all axes, which differs from the previously described data sets. The final preprocessed data set included 21 samples with common 1×1×1 $\textrm{mm}^{3}$ voxel-wise spacing and 128×128×128 voxel space.

At the end of the preprocessing and for all data sets, an image intensity adjustment was performed with a Z-Score normalization per sample. The raw and preprocessed data for all data sets can be seen in Fig. 1a.

### Baseline Training

The cine-CMR and LGE-CMR data sets were split to perform a five-fold cross-validation. For the ex vivo CMR data set, a seven-fold cross-validation was applied by setting the three image-GTL pairs belonging to the same subject (T1, T2, and DWI b0) for validation in every fold. Thus, the training/validation sets consisted of 256/64, 252/63 and 18/3 samples in every fold for the cine, LGE, and ex vivo data sets, respectively.

**Fig. 2:**
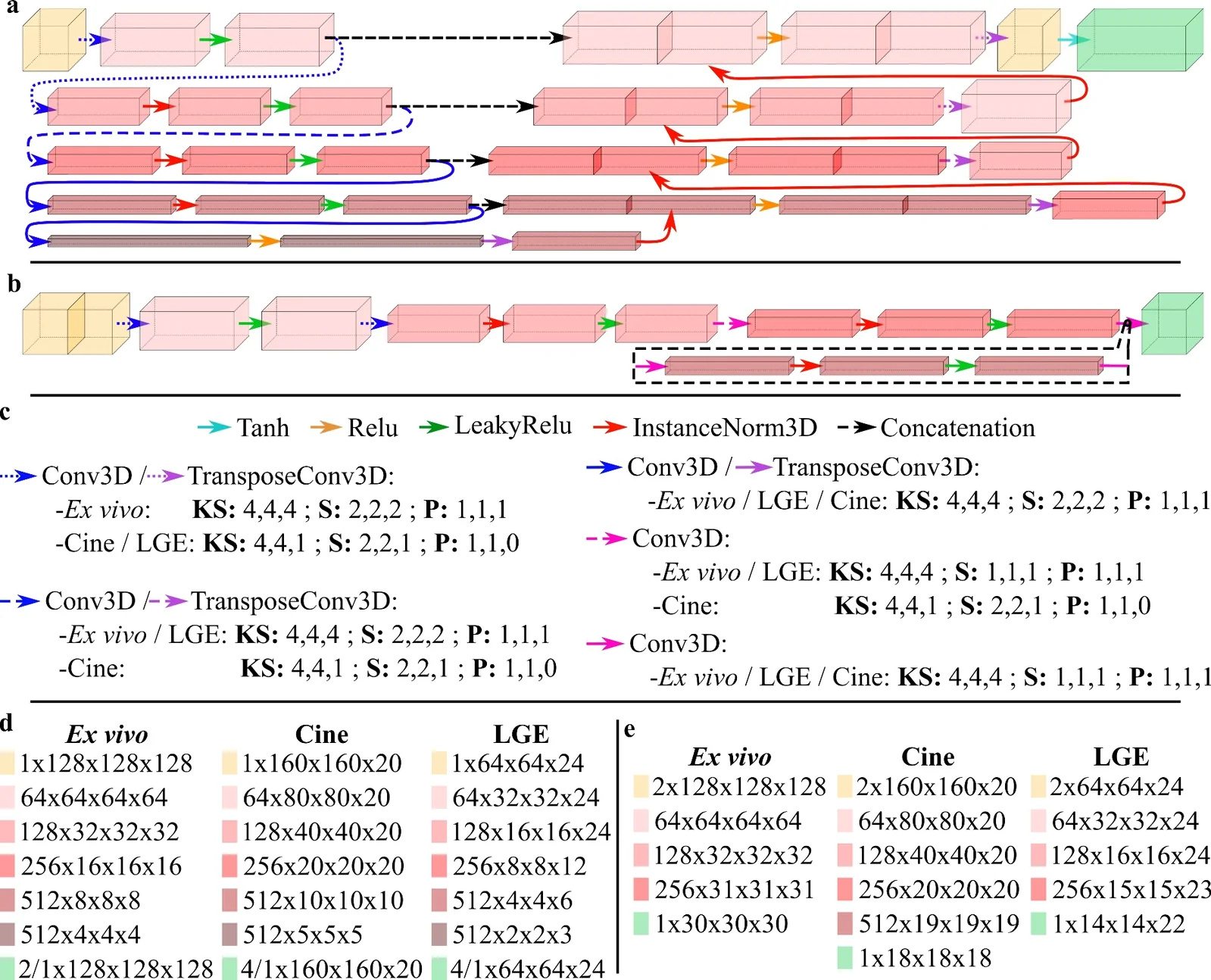
Graphical architectures of the UNet/G (**a**) and D (**b**), showing operations and corresponding feature map shapes. **c** Summary of CNN operations and convolutional layer configurations used across data sets. Mapping of feature map shapes to the color-coded components of the G (**d**) and D (**e**). In (d), note that output classes vary depending on the UNet/G case. *KS* kernel size, *P* padding, *S* stride, *LGE* late gadolinium-enhanced, *G* generator, *D* discriminator, *CNN* convolutional neural network

The 3D UNet architecture [5] shown in Fig. 2a was used for the segmentation of all data sets. Following [4], the outermost 3D convolutions treated the input 3D array as a 2D stack for the highly anisotropic data sets. Namely, when the feature map downsampling in the SAx plane resembled the out-of-plane voxel resolution, the 3D convolutions were applied on the entire 3D feature map as a whole, i.e., the kernel size became isotropic (see Fig. 2a, c, and d).

The CNN was always trained for 250 epochs, and the batch size was chosen to have the same maximum amount of samples per batch, while avoiding memory overload. The batch size was set to three samples for the ex vivo data set and 4 samples for the cine and LGE data sets. Furthermore, the Adam method was used for stochastic optimization with momentum parameters of 0.5 ($\beta _1$) and 0.999 ($\beta _2$). The learning rate was steady at 0.0002 during the first 125 epochs and linearly decreased to 0 in the second half of the training. A cross-entropy loss was selected as the objective function for the optimization problem and both training and validation losses were monitored during training. On-the-fly random standard DA consisted of rotation around LAx (p = 0.4), array flip on the SAx plane (p = 0.5), blurring (p = 0.4), Gaussian noise (p = 0.4), bias field (p = 0.4), and one of motion, spike, and ghost MR artifacts (p = 0.5). Intensity alterations were applied only to the image, while spatial alterations were applied to the image-GTL pair. Then, the array intensity of all samples was normalized to the [− 1,1] range and, only for the samples in the training set, they were shuffled on the fly before being input to the CNN.

The baseline (B) segmentations corresponded to the predictions from this approach, trained without SA (“GAN-Based Data Augmentation” Sect.) and without retraining-mediated TC (“Topological Correction and Priors Definition” Sect.).

### GAN-Based Data Augmentation

This step involved the implementation of a cGAN to generate synthetic images from modified GTLs. The UNet architecture described in “Baseline Training” Sect. was also used here as G. As a mapping from GTL to the image domain was learned, the output array differed in the number of classes with respect to the UNet defined in “Baseline Training” Sect. (see the voxel space of the output array in Fig. 2d). For example, the UNet output in the cine case was 4×160×160×20, since 4 classes were segmented, while when used as G the output array was of 1×160×160×20 for the same data, since a grayscale image was obtained.

The adversarial training of G was achieved with the help of a patch level D (Fig. 2b), as proposed in [15]. Here, D encoded patch-wise information of the real or synthetic image with its GTL. As done for the UNet in “Baseline Training” Sect., an initial 2D encoding was performed in the SAx plane until the feature map became nearly isotropic, then convolutions started to involve inter-slice information. The global evaluation of the latent space defined whether an image was fake or real. As reported in [15], increasing the receptive field of D led to improved representation of high-frequency features on synthetic images. Authors showed that for an isotropic 286-pixel image, it was sufficient to use a 70×70 (24.4% relative) receptive field to capture sharp image details and avoid tiling artifacts. As can be deduced from Fig. 2, here the sizes of the receptive fields were 34×34×34, 70×70×7, and 34×34×7 for the ex vivo, cine, and LGE CMRs, respectively. Thus, the smallest receptive field versus input shape relation obtained here (26.6%) was superior to the one defined in [15], considered to be sufficiently accurate.

The same training samples, epochs, batch sizes, optimizer, and learning rate schedule described in “Baseline Training” Sect. were used to train the GANs. As the input consisted of GTLs instead of images, only the random spatial on-the-fly standard DA was applied to the shuffled training samples. In addition, the intensity on image-GTL pairs was normalized to the [− 1,1] range. The D and G losses were obtained as follows [15]:

1 $$\begin{aligned} G_{loss}= & \text {MSE}(D(\text {GTL},S_i),R) + \lambda \hspace{3pt} \text {L1}(S_i,R_i) \end{aligned}$$

2 $$\begin{aligned} D_{loss}= & 0.5 \hspace{3pt} [ \text {MSE}(D(\text {GTL},S_i), S) + \text {MSE}(D(\text {GTL},R_i), R) ] \end{aligned},$$

where $\text {MSE}$ stands for mean squared error, $\text {L1}$ measures the mean absolute error, $R_i$ and $S_i$ are real and synthetic images, and *R* and *S* are binary arrays that represent completely real and synthetic predictions, respectively. The regularization term λ was set to 100. From these definitions, it follows that when G performs well, the $D_{loss}$ increases while the $G_{loss}$ decreases, and when D performs well, the $D_{loss}$ decreases while the $G_{loss}$ increases, defining this, the adversarial learning.

Once the GAN was trained, the GTLs of the training samples were deformed for synthetic image generation, as shown at the bottom right of Fig. 1a. For cine and ex vivo data, the deformation involved random scaling in the ±20% range, random rotation along LAx, and random translation in the SAx plane in the ±10 mm range. For LGE data, the random scaling and translation ranges were reduced to a half. In addition, non-rigid deformation was applied in the SAx plane with a random elastic deformation with a maximum displacement equivalent to 10% of the LV ED diameter. Finally, the new synthetic data were combined with the training set to define the SA approach.

### Topological Correction and Priors Definition

Once the UNet has been trained, a sample-wise TC can be enforced by retraining the network to minimize a topological loss without substantially changing the original prediction, as reported by Byrne et al. [20]. The baseline training settings were reused for this post-training with the exception of the now steady $1 \times 10^{-5}$ learning rate. Post-training optimization was performed on each validation sample for 100 iterations.

The set of class-wise logits obtained for an input ${\textbf {X}}$ with GTL ${\textbf {Y}}$ was denoted as $\hat{{\textbf {Y}}}^c$ with c=1,2,...,C, where *C* corresponds to the number of foreground classes in the data set. Here, the topological evaluation was applied to the topological classes that corresponded not only to the single foreground classes but also to their combination, as a previous work reported that an improved TC was achieved in that way [20]. $\hat{{\textbf {Y}}}^n$ was defined as the logits of the $n^{th}$ plausible topological class obtained from a single or combined $\hat{{\textbf {Y}}}^c$. In addition, the lifetime of the $l^{th}$ topological structure with dimension *d* found on $\hat{{\textbf {Y}}}^n$ was denoted as $\Delta p_{l,d}(\hat{{\textbf {Y}}}^n)$. If $\Delta p_{d}(\hat{{\textbf {Y}}}^n) = \{ \Delta p_{0,d}(\hat{{\textbf {Y}}}^n),...,\Delta p_{L,d}(\hat{{\textbf {Y}}}^n) \}$ , where $\Delta p_{0,d}(\hat{{\textbf {Y}}}^n)>... > \Delta p_{L,d}(\hat{{\textbf {Y}}}^n)$, with *L* the total amount of *d*-dimensional topological structures found in $\hat{{\textbf {Y}}}^n$, the topological loss was defined as follows:

3 $$\begin{aligned} L_{topo}= & \sum _{d,n} B_{d}^{n} - A_{d}^{n} + Z_{d}^{n} \end{aligned}$$

4 $$\begin{aligned} A_{d}^{n}= & \sum _{l=1}^{B_{d}^{n}} \Delta p_{l,d}(\hat{{\textbf {Y}}}^n) \qquad Z_{d}^{n} = \sum _{l=B_{d}^{n}+1}^{L} \Delta p_{l,d} (\hat{{\textbf {Y}}}^n), \end{aligned}$$

where $B_{d}^{n}$, $A_{d}^{n}$ , and $Z_{d}^{n}$ are the topological prior and the added lifetimes of the correct and incorrect topological structures, respectively, on dimension *d* for the $n^{th}$ topological class. The final sample-wise retraining loss was obtained as the sum of this $L_{topo}$ and the $\text {MSE}$ between the original and the new topologically corrected predictions multiplied by a regularization value of 1000.

**Fig. 3:**
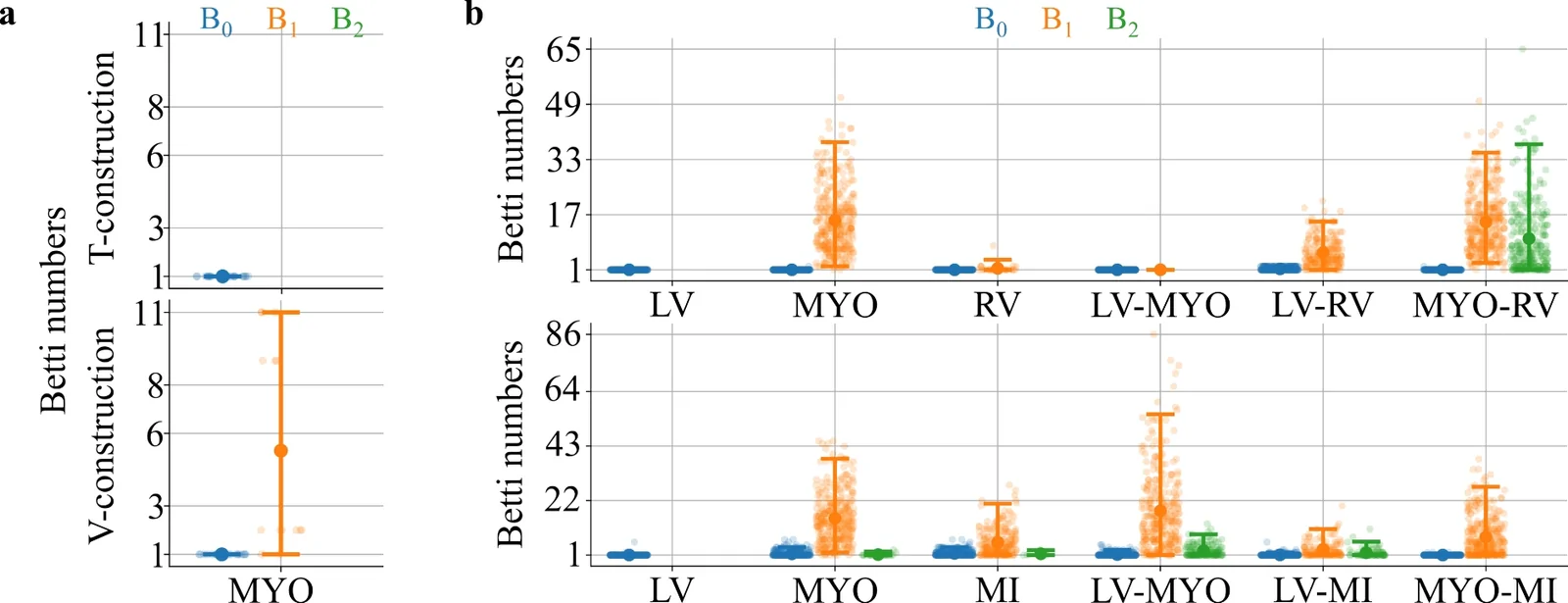
**a** Betti numbers obtained from the preprocessed ex vivo GTLs with V- and T-constructions. **b** Betti numbers for the preprocessed cine (top) and LGE (bottom) CMR data sets along different topological classes. *MYO* myocardium, *LV* left ventricle, *RV* right ventricle, *MI* myocardial infarction, *GTL* ground truth label, *LGE* late gadolinium-enhanced, *CMR* cardiac magnetic resonance, *V/T construction* vertex-/top-cell-based construction

This loss definition shows the importance of selecting topological priors that match the GTL topology. Here, two sources of variation were found in the calculation of Betti numbers: one inherent to the topological construction used for the filtration (Fig. 3a); and the other, inherent to the intrinsic topological discrepancy between samples of the same data set (Fig. 3b). Two cubical complex constructions were evaluated: the vertex-based (V-) construction, in which voxels are assigned to 0-dimensional cells (vertices), and the top-cell-based (T-) construction, in which voxels are assigned to N-dimensional cells (cubes). In 3D, the V-construction defines a 6-neighbor connectivity, whereas the T-construction defines a 26-neighbor connectivity capturing diagonal adjacency between voxels. Specifically, Fig. 3a shows how the V-construction did not lead to null B$_\text {1}$ for the topologically checked ex vivo GTLs, contrary to the T-construction. This occurred because the thin RV apex impaired the V-construction 6-neighbor connectivity to detect the RV as a one-side closed cylinder instead of a tunnel. Thus, all topological calculations for this study were performed with the T-construction. Moreover, we defined the final $B_{d}^{n}$ (F) for a data set by comparing the expected $B_{d}^{n}$ (E) with the mode of the Betti numbers measured (M) in the preprocessed data (see Tables 1 and 2). If E and M were equal, F was set as this value, while if E was different from M, F was set as -1, which means that $L_{topo}$ will not be affected by this prior. The only exception was made for B$_\text {0}$ in the LV-RV topological class, for which F was set at 2.

**Table 1:** Preprocessed cine topological priors (B$_\text {0}$, B$_\text {1}$, B$_\text {2}$)

	LV	MYO	RV	LV-MYO	LV-RV	MYO-RV
E	(1,0,0)	(1,1,0)	(1,0,0)	(1,0,0)	(2,0,0)	(1,1,0)
M	(1,0,0)	(1,7,0)	(1,0,0)	(1,0,0)	(1,0,0)	(1,7,0)
**F**	**(1,0,0)**	**(1,-1,0)**	**(1,0,0)**	**(1,0,0)**	**(2,0,0)**	**(1,-1,0)**

*E* expected priors, *M* measured priors, *F* final priors, *LV* left ventricle, *MYO* myocardium, *RV* right ventricle

**Table 2:** Preprocessed LGE topological priors (B$_\text {0}$, B$_\text {1}$, B$_\text {2}$)

	LV	MYO	MI	LV-MYO	LV-MI	MYO-MI
E	(1,0,0)	(1,1,0)	(1,0,0)	(1,0,0)	(1,0,0)	(1,1,0)
M	(1,0,0)	(1,7,0)	(1,0,0)	(1,7,0)	(1,0,0)	(1,1,0)
**F**	**(1,0,0)**	**(1,-1,0)**	**(1,0,0)**	**(1,-1,0)**	**(1,0,0)**	**(1,1,0)**

*E* expected priors, *M* measured priors, *F* final priors, *LV* left ventricle, *MYO* myocardium, *MI* myocardial infarction, *LGE* late gadolinium-enhanced

### Inference and Postprocessing

After calculating the predictions, we brought all samples to their original physical space, with original spacing and voxel space, by reversing the preprocessing steps of cropping, padding, and resampling. Next, every segmentation approach was evaluated by calculating overlapping and topological similarity metrics. Prediction and GTL class-wise spatial overlap and contour distance were assessed with the widely known per-class Dice Similarity Score (DSC) and Hausdorff Distance (HD) metrics. Complementary and as defined by Byrne et al. [20], the topological correctness between prediction and GTL was assessed by computing the absolute difference between Betti numbers, named Betti error (BE), per class. In instances where a specific class was present in the GTLs but absent in the model’s prediction (false negatives), the HD could not be computed. To account for these cases and ensure a comprehensive evaluation, we separately report the frequency of false negative occurrences. Furthermore, results for different approaches were compared using the non-parametric Wilcoxon signed-rank test with Benjamini–Hochberg correction.

**Fig. 4:**
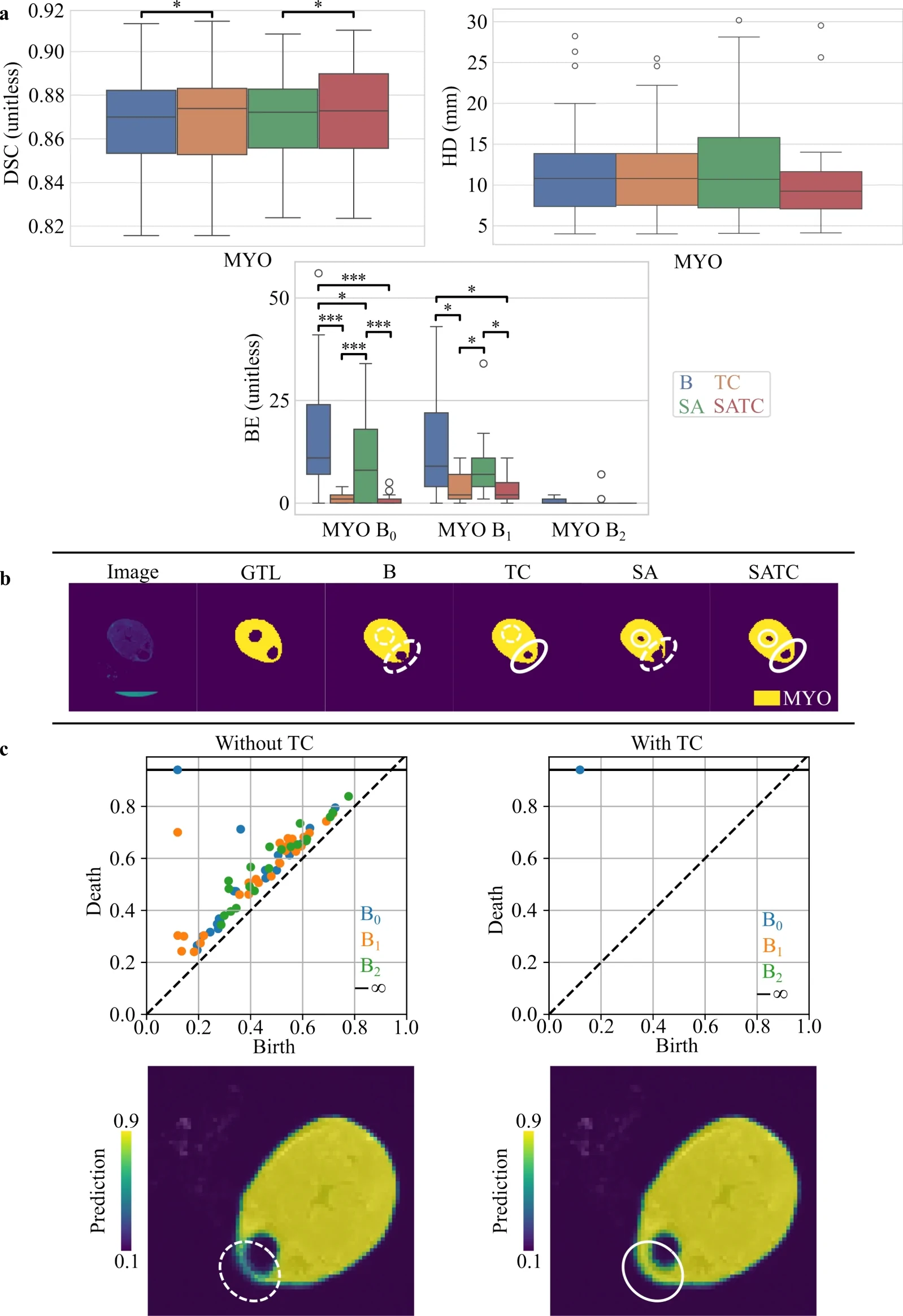
Segmentation performance on the ex vivo data set. **a** Quantitative DSC (left), HD (right), and BE (bottom) outcomes. The statistical significance of the difference between the two distributions is indicated by *p< 0.05, **p< 0.01, and ***p< 0.001. **b** Qualitative comparison highlighting the improved delineation of the RV and LV apical regions obtained with the different methodologies. Dashed and solid white contours denote regions of poor and improved segmentations, respectively. **c** Topological analysis: Persistence lifetime plots (top) and logit maps near the RV apex (bottom) for B (left) and TC (right) approaches. In persistence lifetime plots, topological features located near and far from the diagonal (i.e., with shorter and longer lifetimes) correspond to spurious and robust topological structures encountered throughout the PH filtration of the prediction. When TC is applied (top-right graph), the 3D prediction correctly exhibits a single persistent connected component, yielding the expected topology (B_0_ = 1, B_1_ = 0, B_2_ = 0; see “Ex Vivo CMR” Sect.). *DSC* Dice similarity score, *HD* Hausdorff distance, *BE* Betti error, *MYO* myocardium, *B* baseline, *TC* topological correction, *SA* synthetic augmentation, *SATC* synthetic augmentation and topological correction, *GTL* ground truth label, *LV* left ventricle, *RV* right ventricle, *CMR* cardiac magnetic resonance, *PH* persistent homology, *3D* three dimensional

## Results

After training, final predictions were generated for the B, SA, TC, and combined SA and TC (SATC) approaches. The results for these four approaches across all data sets are presented below.

### *Ex Vivo* Data Set

The results of the analysis for the *ex vivo* data set are depicted in Fig. 4. In terms of median DSC, both TC and SA produced slightly higher values of 87.4% (p< 0.05) and 87.2% (non-significant), respectively, compared to 87% for case B (top-left panel in Fig. 4a). The addition of synthetic images not only improved the median DSC but also reduced its dispersion by almost 11%. The SATC approach maintained the improvements achieved with SA, while significantly increasing the median DSC from 87.2% to 87.3%.

In terms of BE (bottom panel in Fig. 4a), the largest error was found for connected components and tunnels. The median BE values were 11, 1, 8, and 0 in B$_\text {0}$, and 9, 2, 7, and 2 in B$_\text {1}$ for approaches B, TC, SA, and SATC, respectively. These results showed a major, statistically significant BE reduction at the B$_\text {0}$ topology, when topological-aware segmentation (p< 0.001) and GAN-based DA (p< 0.05) were performed. In this line, statistically significant decrease in topological error was observed when TC was used in combination with B or SA approaches. In addition, 2 and 4 samples were segmented with exact topology when using TC and SATC, respectively.

Figure 4b qualitatively illustrates how SA and TC contribute to improved segmentation. TC correctly closed the RV at the apex, thereby preventing the formation of an artificial tunnel, while SA avoided premature closure of the LV blood pool. RV closure is particularly challenging in automatic segmentation due to its extremely thin myocardial wall at the apex. As shown in Fig. 4c, the B logits were refined by TC. This behavior is further clarified in the persistence lifetime plots. In these diagrams, topological features located near and far from the diagonal (i.e., with shorter and longer lifetimes) correspond to spurious and robust topological structures that persist throughout the PH filtration of the prediction, respectively (top-left graph in Fig. 4c). When TC was applied, it eliminated all spurious non-persistent topologies (top-right graph in Fig. 4c). Thus, the 3D biventricular prediction correctly exhibits a single persistent connected component, consistent with the expected topology (B_0_ = 1, B_1_ = 0, B_2_ = 0; see “Ex Vivo CMR” Sect.).

**Fig. 5:**
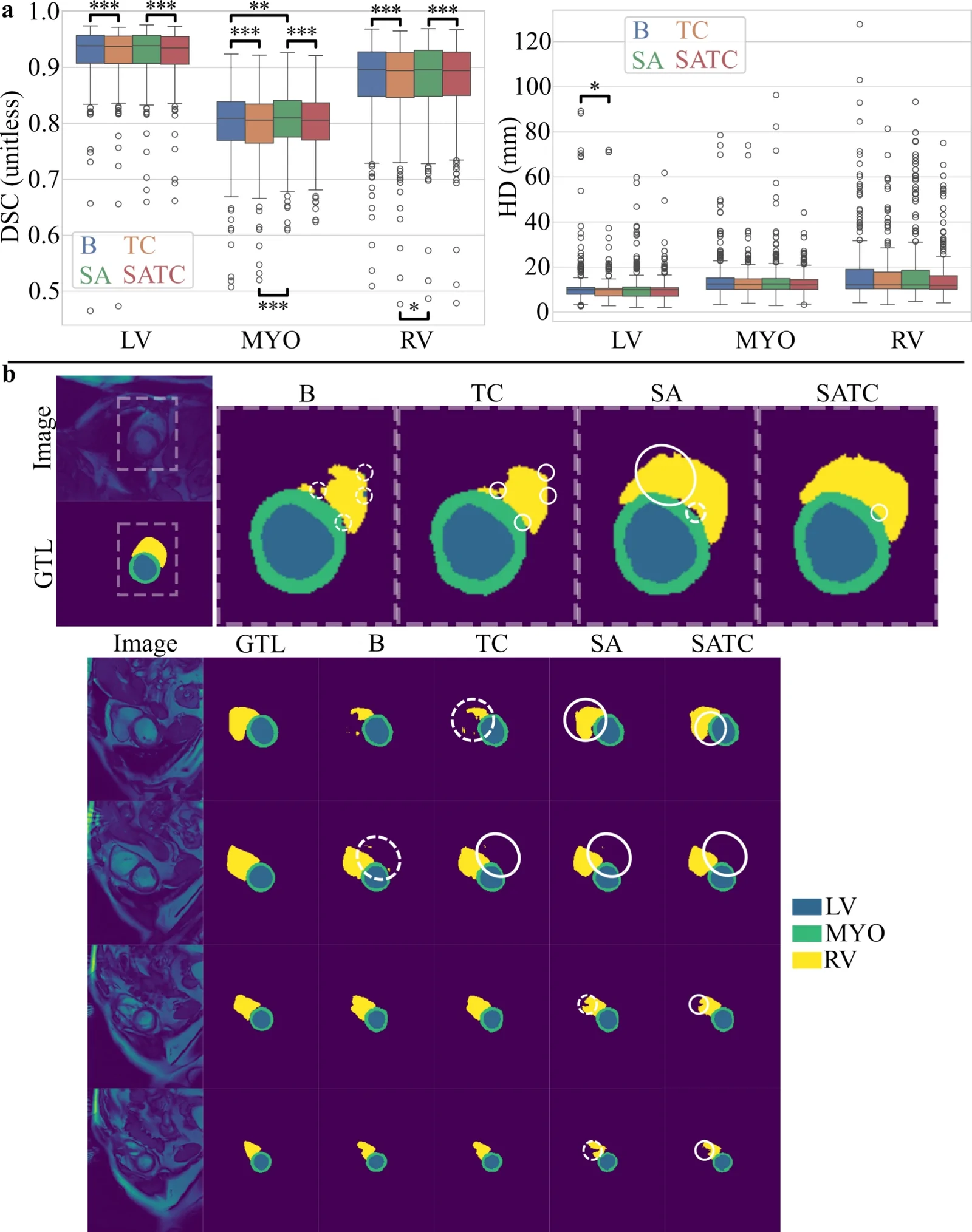
Segmentation performance on the cine data set. **a** Quantitative DSC (left) and HD (right) outcomes. The statistical significance of the difference between the two distributions is indicated by *p< 0.05, **p< 0.01, and ***p< 0.001. **b** Qualitative comparison highlighting the improved segmentation obtained with the different methodologies. Dashed and solid white contours denote regions of poor and improved segmentations, respectively. *DSC* Dice similarity score, *HD* Hausdorff distance, *MYO* myocardium, *LV* left ventricle, *RV* right ventricle, *B* baseline, *TC* topological correction, *SA* synthetic augmentation, *SATC* synthetic augmentation and topological correction, *GTL* ground truth label

**Fig. 6:**
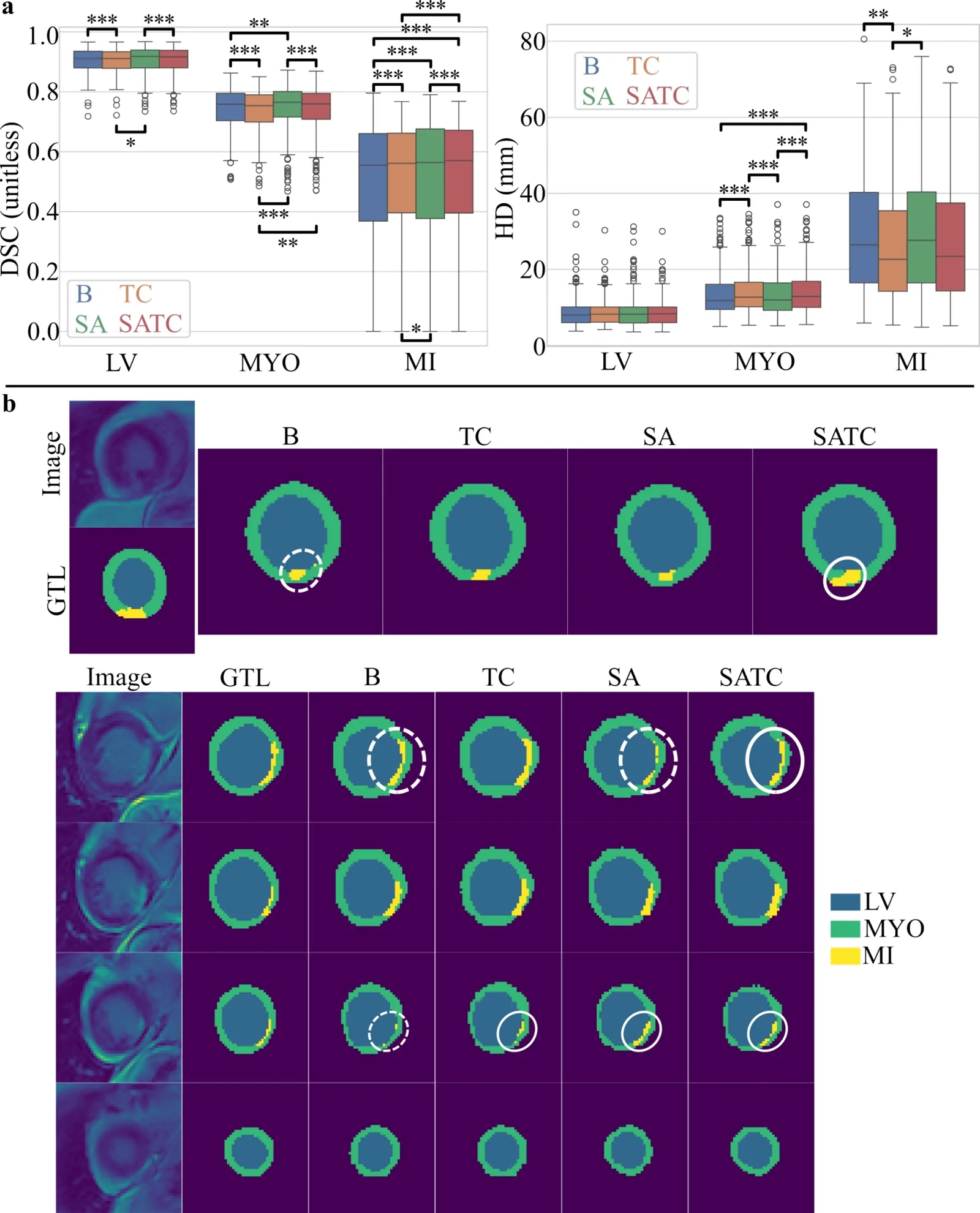
Segmentation performance on the LGE data set. **a** Quantitative DSC (left) and HD (right) outcomes. The statistical significance of the difference between the two distributions is indicated by *p< 0.05, **p< 0.01, and ***p< 0.001. **b** Qualitative comparison highlighting the improved segmentation obtained with the different methodologies. Dashed and solid white contours denote regions of poor and improved segmentations, respectively. *LGE* late gadolinium-enhanced, *DSC* Dice similarity score, *HD* Hausdorff distance, *MYO* myocardium, *LV* left ventricle, *MI* myocardial infarction, *B* baseline, *TC* topological correction, *SA* synthetic augmentation, *SATC* synthetic augmentation and topological correction, *GTL* ground truth label

### Cine Data Set

Figure 5 presents the results for the cine data set, which showed minimal variations in the multi-class DSC and HD values for all approaches. Statistically significant (p< 0.001) DSC reductions of up to 0.4% compared to the B approach were observed for all classes when TC-based approaches were used. Slight increments in DSC were found for SA with respect to B. These DSC increments were statistically significant for the MYO (p<0.01) class (see left panel in Fig. 5a). The median HD remained stable when the different approaches were used for the evaluation of the LV. For the MYO and RV classes, the median HD was minimally reduced by 1.78% and 0.09%, respectively, for TC with respect to B. SATC led to additional median decreases in HD with respect to B for the same MYO (2.7%) and RV (2.3%) classes. None of the median HD differences across approaches for the MYO and RV classes reached statistical significance (right panel in Fig. 5a).

**Fig. 7:**
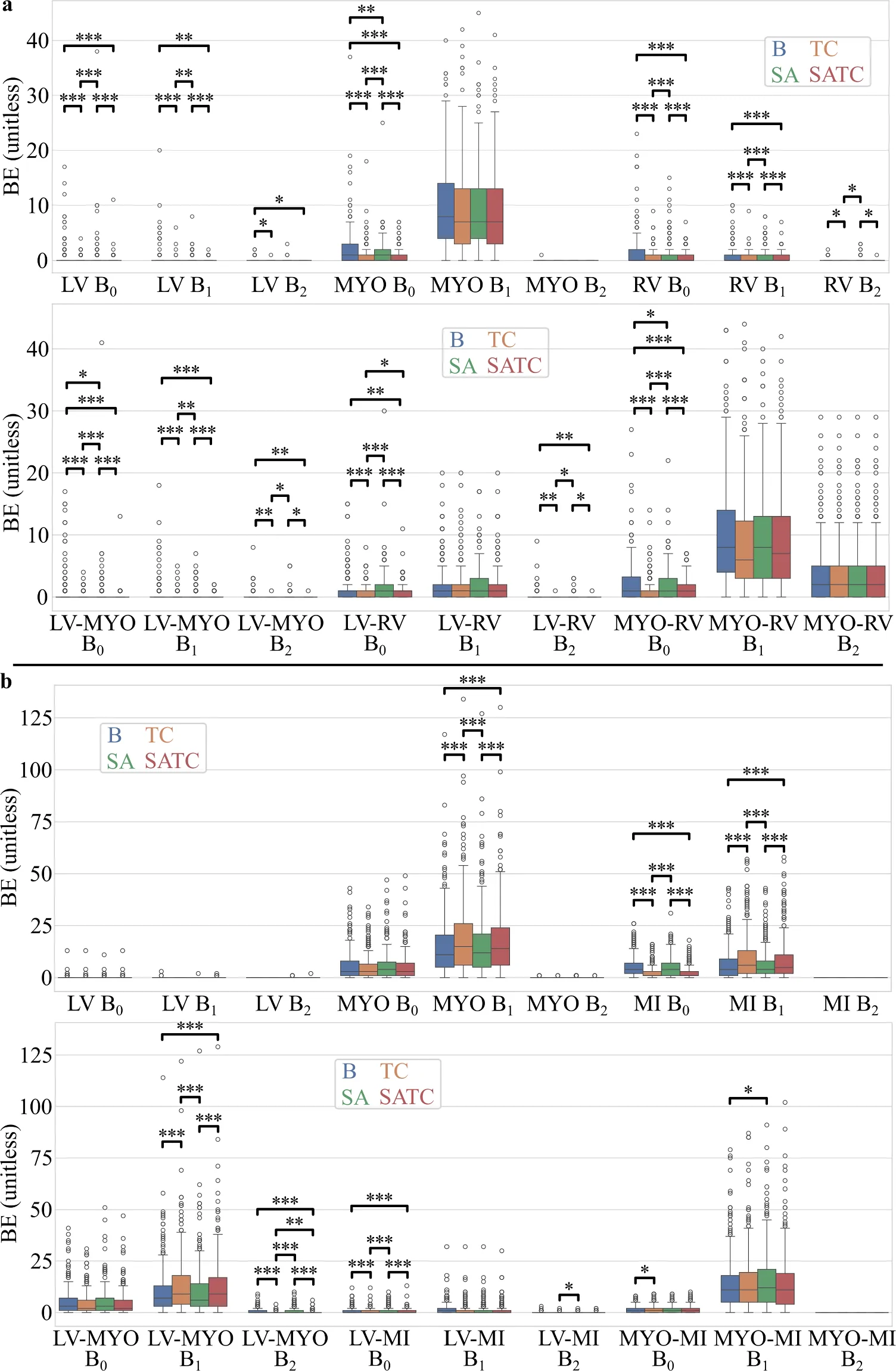
Computed BE for all topological classes (individual: top and combined: bottom) in the cine (**a**) and LGE (**b**) data sets. The statistical significance of the difference between the two distributions is indicated by *p< 0.05, **p< 0.01, and ***p< 0.001. *BE* Betti error, *MYO* myocardium, *LV* left ventricle, *RV* right ventricle, *MI* myocardial infarction, *B* baseline, *TC* topological correction, SA synthetic augmentation, SATC: synthetic augmentation and topological correction.

As for the *ex vivo* data set, the highest BE values were found for the Betti numbers B$_\text {0}$ and B$_\text {1}$ (Fig. 7a). In the MYO class, the lowest, statistically significant B$_\text {0}$-related BE values were found when TC and SA were applied (B-TC: p< 0.001, B-SA: p< 0.01, B-SATC: p< 0.001). The maximum values of B$_\text {0}$-related BE in the MYO class were 37, 18, 25, and 7 for the segmentation obtained using B, TC, SA, and SATC. Furthermore, the median B$_\text {1}$-related BE decreased by 1 in this class for all other approaches with respect to B. In the RV class, when the Betti numbers B$_\text {0}$ and B$_\text {1}$ were analyzed, the maximum BE values of 23 and 10 obtained for the B approach decreased to 7 for SATC. For this class and Betti numbers, the BE distributions were statistically different when TC was employed (B-TC: p< 0.001, B-SATC: p< 0.001). The B$_\text {0}$- and B$_\text {1}$-related BE dispersion followed the same trend for the approaches tested. In the combined LV-RV topological class, the 75$^{th}$ percentile for B$_\text {0}$- and B$_\text {1}$-related BE took the highest value when the SA approach was used. The maximum BE values found for B (27) and SA (22) were significantly reduced for TC (14) and SATC (7), when B$_\text {0}$ was assessed in the MYO-RV topological class. In the same topological class but for B$_\text {1}$, the median BE decreased from B to TC by 2 and from SA to SATC by 1. The highest BE values were found for the MYO and MYO-RV topological classes in B$_\text {1}$.

Figure 5b illustrates the corrections made in the B predictions by TC, SA, and SATC in two cardiac CMRs. The addition of synthetic images improved the RV segmentation while TC erased spurious connected components of all classes and closed the erroneous space between the MYO and RV classes.

### LGE Data Set

As shown in Fig. 6a, TC minimally reduced the median DSC values with respect to B in the LV (0.1%, p< 0.001) and MYO (0.7%, p< 0.001) classes. SA slightly increased the median DSC with respect to B by 0.64% (without statistical significance) and 0.86% (p< 0.01) for the LV and MYO classes, respectively. SATC minimally reduced the increase in median DSC induced by SA for LV and MYO (p< 0.001). In the MI class, the median DSC increased steadily by 1.1, 1.7, and 2.8% when TC, SA, and SATC were employed (p< 0.001). The right panel in Fig. 6a shows how the median HD changed minimally from 8 mm for B to 8.3 mm for SATC in LV segmentation. In the MYO class, TC unexpectedly increased the median HD found in B by 7.4% for TC and 9% for SATC (p< 0.001). The opposite behavior was observed in MI segmentation, where a decrease in B of 14.4% (p< 0.01) and 11.5% (without statistical significance) in the median HD was calculated when TC and SATC were used, respectively. Importantly, the use of topological correction resulted in false-negative segmentations of the MI class in 10 and 3 samples for the TC and SATC approaches, respectively, whereas no false negatives were observed with the B and SA approaches.

Here, as in the cine data, the highest BE values were found for the topologies B$_\text {0}$ and B$_\text {1}$, especially in the MYO and MI classes, as can be seen in Fig. 7b. For MI, the maximum BE for the topology B$_\text {0}$ was reduced by 10 (median BE decreased by 3, p< 0.001) and 13 (median BE decreased by 3, p< 0.001) when TC and SATC were used compared to B and SA, respectively. The maximum BE for the topology B$_\text {1}$ was increased by 14 (median 2, p< 0.001) and 15 (median 1, p< 0.001) with respect to B and SA when TC and SATC were used for MI segmentation. Moreover, regarding the generation of spurious tunnels in the MYO class, it can be observed that TC increased BE, with relative median increments of 4 and 2 for the B-TC (p< 0.001) and SA-SATC (p< 0.001) pairs, respectively. When comparing the LV-MYO class against the MYO class, the median number of spurious tunnels was lower in all four approaches for the LV-MYO class (reduction of B:4, TC:6, SA:6, and SATC:5 from MYO to LV-MYO). However, the trend of having higher number of erroneous tunnels on TC and SATC if compared with B and SA remained (p< 0.001). When the MYO class was combined with the MI class, TC did not increase the median BE with respect to B for the topology B$_\text {1}$. SA presented a median number of erroneous tunnels that was higher by 1 than all other approaches.

As qualitatively presented in Figure 6b and quantitatively illustrated by the measures of DSC and HD, TC, and SA contributed to improved precision in the segmentation of the MI class.

## Discussion

Data scarcity and data variability are common problems in CMR segmentation that hamper the performance of DL algorithms. Here, we assessed the usage of GAN-based DA and TC in several CMR data sets presenting these drawbacks. Lately, GAN-based DA and PH have shown some ability to assist in various segmentation tasks individually. To the best of our knowledge, this is the first time that their combination has been tested on the 3D segmentation of several complex multi-contrast CMR data sets.

Most modern CMR data sets lack strict 3D topological consistency, as evidenced by the large dispersion in Betti numbers shown in Fig. 3b. Two compounding factors underlie this limitation. First, out-of-plane slice misalignment in in vivo, anisotropic cine, and LGE acquisitions introduces inherent geometric discontinuities between slices. Second, and more fundamentally, annotators have historically focused on the precise delineation of individual slices, while overlooking 3D topological coherence, a practice reinforced by the limited awareness of PH and the unavailability of computationally efficient tools for its calculation until recently [28]. Together, these factors produce large Betti number dispersion in thin, non-bulky structures such as the MYO and MI classes and, more critically, can yield topologically unrealistic GTLs in anisotropic, misaligned data sets. Such inconsistencies further propagate to related topological classes, such as LV-RV in the cine data, and ultimately undermine the effectiveness of TC. Critically, the topological priors used in this work are fixed at the data set level, i.e., a single set of Betti numbers is assigned per topological class across all samples. When GTL topological profiles are neither consistent on a per-case basis nor uniform across samples, such a fixed prior becomes an inadequate and potentially misleading supervisory signal, which explains the contained but non-negligible performance deterioration of TC under these conditions, specially for GTL-prediction overlapping metrics [20]. To mitigate this, we deliberately avoided imposing a fixed topological prior for topological classes exhibiting high inter-sample topological variability (see “Topological Correction and Priors Definition” Sect.), which helped contain performance deterioration. To further demonstrate that TC achieves strong performance when GTL topology is coherent and well defined, we constructed a 3D *ex vivo* data set with isotropic voxel space and voxel-wise spacing, and controlled GTL topology, to rigorously evaluate TC applied to a CNN trained on topologically consistent data.

In the work presented by Byrne et al. [20], the authors showed that, in terms of DSC, TC greatly deteriorated the 3D segmentation of the whole heart, while, in terms of HD, the segmentation of all classes improved with respect to baseline. Here, TC led to small (lower than 0.7%) reductions or increments in DSC across all 3D data sets. The more contained deterioration observed here compared to Byrne et al. [20] is likely attributable to our minimally enhanced topological loss formulation, in which we avoided imposing priors on topological features found to be highly inconsistent across samples in the data set (see “Topological Correction and Priors Definition” Sect.). Generally, TC reduced HD across data sets. The largest reduction was observed in the MI class of the LGE data, demonstrating that a data set-level topological prior can still produce overall improvements in HD despite substantial inter-sample topological heterogeneity, provided that highly variable topological features are excluded from guiding the PH-based retraining of the CNN. However, an unexpected increase in median HD was observed for the MYO class when TC was applied to LGE data, a deterioration not reproduced in the cine data. We attribute this to the additional topological variability introduced by heterogeneous MI substrates in LGE, which creates a systematic mismatch between the fixed, data set-level priors, and the sample-specific anatomy. This is supported by the higher Betti number dispersion in MYO and MYO-related topological classes in LGE compared to cine (Fig. 3b), and more importantly, by the increase in BE observed when TC was applied to LGE MYO classes, which was not observed when TC was applied to cine MYO classes (Fig. 7). This particular case illustrates a fundamental limitation of fixed topological priors: when inter-sample anatomical variability is high, as in LGE MYO due to variable MI extent and distribution, enforcing a uniform topological constraint can conflict with sample-specific topology and degrade spatial accuracy. Except for this case, TC generally produced contained changes in DSC and modest HD improvements across topologically inconsistent data sets. Importantly, the analysis of *ex vivo* CMRs confirmed that topological consistency in the GTLs is a prerequisite for maximizing the benefits of TC, not only in terms of topological correctness but also in spatial overlap between prediction and GTL. The suppression of all but the largest connected components is a common postprocessing practice in modern segmentation pipelines, which we deliberately avoided here, as Byrne et al. [20] have already demonstrated that TC surpasses this technique in topological correctness. Altogether, these results highlight the need for clinicians to account for 3D topological consistency when building new data sets.

The anisotropic data sets comprised a large number of highly variable samples, in contrast to the *ex vivo* data set, which contains fewer samples but with low variability. This property of the *ex vivo* data set made learning feasible with limited data and enabled precise assessment of TC effects. Nevertheless, applying TC with a fixed set of Betti numbers to anisotropic, topologically heterogeneous samples remains a limitation of this work, and directly contributes to the reduction of TC performance described above for the MYO class of the LGE data set. Thus, a natural continuation of this work would involve: first, correction of slice misalignment in the LGE and cine data sets; second, assessment of whether more homogeneous per-class topological profiles are achieved post-alignment; and third, exploration of sample-adaptive topological priors that account for inter-individual anatomical variability, which could substantially improve TC performance in heterogeneous clinical data sets. In this context, unseen samples could be handled in two ways: (i) semi-automatically, by allowing expert-guided manual selection of appropriate topological priors, or (ii) fully automatically, by leveraging the complete set of topological priors observed in the topologically variable GTLs to guide PH-based CNN retraining. The resulting segmentations could then be compared (using, for example, DSC and HD) and the prior yielding the best performance selected as the most suitable topological descriptor for the specific case.

Generally, GAN-based DA slightly improved BE, HD, and DSC in all data sets. Although the overall enhancement was minor, some important improvements were achieved. In the *ex vivo* data set, SA consistently contributed to the reduction of BE. In the LGE data set, the use of SA increased DSC in all classes. In the work of Al Khalil et al. [13], a 2D segmentation pipeline was presented involving GAN-based DA and applied in an out-of-domain context to the same cine data set used here. The improvement achieved by their proposed method with respect to the baseline was reported to be more relevant in terms of DSC and HD metrics than ours. However, the DSC values after SA in that paper [13] (LV: 92.5%, MYO: 82.1%, RV: 90.1%) were very similar to the DSC values obtained here for both B and SA (LV: 93.9%, MYO: 81%, RV: 89.6%). Furthermore, our improvements in terms of DSC when GAN-based DA was used in the LGE case were in agreement with the results in [14]. In that study, the authors reported a 3% increase in DSC for the MI class when comparing the baseline 2D nnUNet with its GAN-augmented counterpart. Thus, good agreement was found between our results, when SA was applied to cine and LGE data sets, and state-of-the-art DL approaches [13, 14], even though different data, preprocessing, CNNs, and more importantly, GAN architectures were used. In this context, these recent studies involving CMR segmentation have suggested that the SA approach proposed here could have achieved better results if a SPADE-GAN architecture had been used, as it mitigates the loss of semantic information typically caused by modern normalization techniques [13, 14, 17]. However, even if suboptimal and taking into account that our baseline results were in the range of cutting-edge algorithms, our GAN-based DA led to small but significant improvements in some of the performance metrics for all data sets tested. This stressed the capacity of GAN-based DA to improve predictions in different CMR data sets. In this line, future studies could be conducted to provide a proper quantitative assessment of how a SPADE GAN could improve segmentation results.

From a practical standpoint, it is important to interpret the modest DSC improvements and the limited size of the *ex vivo* data set in context. First, baseline CNN performance in the cine data set was already high as previously mentioned, leaving limited margin for further gains in overlap-based metrics such as DSC. In such regimes, even small absolute changes may reflect meaningful structural refinements that are not fully captured by global overlap measures, but might impact topological (BE) metrics. This occurred for SA in the *ex vivo* data set (slightly improved DSC but significantly enhanced BE). More importantly, since TC is primarily designed to enforce anatomical plausibility and topological correctness rather than maximize voxel-wise overlap, its practical value lies in stabilizing structural consistency while maintaining competitive DSC performance. Second, although the *ex vivo* data set comprises only 21 samples and does not allow broad generalization, its role in this study was to provide a controlled isotropic setting to isolate the effect of topological consistency on TC performance. The consistent improvements observed in this controlled environment support the mechanistic validity of the approach, while the results in larger, heterogeneous clinical data sets demonstrate its feasibility under realistic conditions. Together, these findings suggest that the practical significance of the proposed framework lies not in large DSC gains alone, but in achieving anatomically coherent segmentations without compromising standard performance metrics.

Although the UNet remains the backbone of most modern CNN-based segmentation systems, more recent frameworks such as nnUNet have demonstrated superior performance [4]. To investigate how the SA and TC strategies proposed in this work interact with a stronger baseline, we trained a 3D full-resolution nnUNet for 250 epochs and evaluated the same SA, TC, and SATC configurations against the baseline case (B) on the LGE data set using DSC and HD. As shown in Table 3, the baseline nnUNet achieved higher performance than our UNet architecture, as expected. Specifically, median DSC (%) for nnUNet was LV: 93.27, MYO: 80.77, MI: 67.21, compared with LV: 91.24, MYO: 75.87, MI: 55.5 for our UNet. Similarly, median HD (mm) improved from LV: 8, MYO: 11.82, MI: 26.5 (UNet) to LV: 6.46, MYO: 10.93, MI: 22.28 (nnUNet). When TC and SA were applied individually or in combination, only contained increments or decrements in performance were observed (Table 3) and a modest amount of false-negative MI segmentations were observed (B: 0, TC: 2, SA: 3, SATC: 5). Importantly, a significant reduction in median HD for the MI class was obtained whenever TC was incorporated (B: 22.28, TC: 17.63, SA: 19.73, SATC: 17.82). This consistent HD improvement in the MI class aligns with the behavior observed using our baseline UNet. Although TC produced a slight (non-significant) increase in DSC (B: 67.21, TC: 67.48), the use of SA led to a small DSC decrease (SA: 66.8; SATC: 67.05) in the MI class, which contrasts with the improvements reported by Lustermans et al. [14] and our results herein. This discrepancy suggests that, for consistently improving spatial overlap in the MI class of LGE data sets, more advanced generative strategies, such as a SPADE-GAN architecture combined with a cascade segmentation pipeline, may be required. Future work should further explore this direction by evaluating the integration of SPADE-GAN-based SA and TC within the nnUNet framework using both the cine and LGE data sets. After addressing slice misalignment and GTL topological inconsistencies, such an evaluation would enable a more rigorous assessment of the interaction between advanced segmentation backbones and topology-aware, synthetically augmented data-driven training strategies.

**Table 3:** Segmentation results obtained when the TC and SA were individually and in combination applied to the nnUNet on the LGE data set

	DSC (%) ↑			HD (mm) ↓		
	LV	MYO	MI	LV	MYO	MI
B	93.27_(90.7, 95.4)_	80.77_(76.2, 83.6)_	67.21_(53.0, 74.0)_	6.46_(5.3, 9.0)_	10.93_(8.0, 14.9)_	22.28_(12.2, 37.0)_
TC	${\textbf {93.25}}_{\textbf {(90.7, 95.4)}}$	$\textit{80.70}_{\textit{(76.1, 83.6)}}$	67.48_(53.4, 73.8)_	6.44_(5.4, 9.0)_	11.05_(8.2, 15.1)_	$\textit{17.63}_{\textit{(10.5, 29.5)}}$
SA	93.17_(90.4, 95.5)_	80.98_(76.1, 83.6)_	${\textbf {66.80}}_{\textbf {(51.8, 73.9)}}$	6.44_(5.3, 9.2)_	10.74_(8.2, 15.2)_	19.72_(11.5, 34.0)_
SATC	${\textbf {93.18}}_{{\textbf {(90.4, 95.5)}}}$	80.82_(76.2, 83.6)_	${\textbf {67.05}}_{{\textbf {(51.9, 73.8)}}}$	6.38_(5.3, 8.9)_	11.09_(8.3, 15.2)_	${\textbf {17.82}}_{{\textbf {(10.5, 30.2)}}}$

Values are presented as $\mathrm{P}_{50(\mathrm{P}_{25},\mathrm{P}_{75})}$. Values in bold and italic indicate statistically significant differences compared to the B approach (bold: p< 0.05; italic: p< 0.01). *LGE* late gadolinium-enhanced, *DSC* Dice similarity score, *HD* Hausdorff distance, *MYO* myocardium, *LV* left ventricle, *MI* myocardial infarction, *B* baseline, *TC* topological correction, *SA* synthetic augmentation, *SATC* synthetic augmentation and topological correction

Overall, the combination of SA and TC demonstrated the capacity to improve segmentation performance. For instance, in MI segmentation on LGE data, the combined SATC approach achieved the best DSC and HD scores: SA increased DSC, while TC corrected the HD errors introduced by SA without compromising DSC. Furthermore, in the *ex vivo* data set, applying TC after SA led to reductions in HD and BE, along with an increase in DSC, compared with the baseline scenario in which neither TC nor SA was applied. In this data set, the combined use of SA and TC yielded a greater reduction in BE than either method alone. Notably, improvements in prediction overlap with the GTL were predominantly observed when both approaches were applied jointly rather than separately in the *ex vivo* data set. In conclusion, we evaluated how GAN-based DA and topology-driven corrections can enhance 3D CNN-based segmentation of complex multi-vendor, multi-center, multi-class, and multi-contrast CMR data. For the isotropic, topologically homogeneous *ex vivo* data set, both SA and TC, applied individually or in combination, resulted in clear and consistent segmentation improvements. In the cine and LGE data sets, modest yet significant improvements were achieved despite low out-of-plane resolution and inter-slice misalignment, which introduced topological heterogeneities. Importantly, the combination of SA and TC led to markedly improved infarction segmentation in the LGE data. Altogether, these findings underscore the potential of the proposed methods for CMR segmentation and justify their integration and further study within state-of-the-art segmentation frameworks.

## Data Availability

The full source code for this work is available at https://github.com/lino202/3Dseg, while the nnUNet implementation with topological correction is available at https://github.com/lino202/nnUNet. The CMR images used are available from the M&Ms challenge (https://www.ub.edu/mnms/), the Emidec challenge (https://emidec.com/), the MYOSAIQ challenge (https://www.creatis.insa-lyon.fr/Challenge/myosaiq/index.html), and a porcine DW-CMR data set (https://med.stanford.edu/cmrgroup/data/ex_vivo_dt_mri.html).
